# Chronic Caffeine Treatment Protects Against α-Synucleinopathy by Reestablishing Autophagy Activity in the Mouse Striatum

**DOI:** 10.3389/fnins.2018.00301

**Published:** 2018-05-02

**Authors:** Yanan Luan, Xiangpeng Ren, Wu Zheng, Zhenhai Zeng, Yingzi Guo, Zhidong Hou, Wei Guo, Xingjun Chen, Fei Li, Jiang-Fan Chen

**Affiliations:** ^1^Molecular Neuropharmacology Laboratory, School of Optometry and Ophthalmology and Eye Hospital, Wenzhou Medical University, Wenzhou, China; ^2^State Key Laboratory of Optometry and Vision Science, Wenzhou, China; ^3^Department of Neurology, Boston University School of Medicine, Boston, MA, United States

**Keywords:** α-synuclein, caffeine, autophagy, macroautophagy, α-synucleinopathy, Parkinson's disease, striatum

## Abstract

Despite converging epidemiological evidence for the inverse relationship of regular caffeine consumption and risk of developing Parkinson's disease (PD) with animal studies demonstrating protective effect of caffeine in various neurotoxin models of PD, whether caffeine can protect against mutant α-synuclein (α-Syn) A53T-induced neurotoxicity in intact animals has not been examined. Here, we determined the effect of chronic caffeine treatment using the α-Syn fibril model of PD by intra-striatal injection of preformed A53T α-Syn fibrils. We demonstrated that chronic caffeine treatment blunted a cascade of pathological events leading to α-synucleinopathy, including pSer129α-Syn-rich aggregates, apoptotic neuronal cell death, microglia, and astroglia reactivation. Importantly, chronic caffeine treatment did not affect autophagy processes in the normal striatum, but selectively reversed α-Syn-induced defects in macroautophagy (by enhancing microtubule-associated protein 1 light chain 3, and reducing the receptor protein sequestosome 1, SQSTM1/p62) and chaperone-mediated autophagy (CMA, by enhancing LAMP2A). These findings support that caffeine—a strongly protective environment factor as suggested by epidemiological evidence—may represent a novel pharmacological therapy for PD by targeting autophagy pathway.

## Introduction

Parkinson's disease (PD) is pathologically characterized by selective degeneration of dopaminergic neurons in the midbrain and the presence of intracellular amyloidogenicα-synuclein (α-Syn) inclusions, known as Lewy bodies and Lewy neurites, which progress from the medulla to midbrain and throughout cortical areas. The studies over the last two decades have identified altered α-Syn function as a key molecular pathogenesis mechanism of PD (Goedert, 2001; Lashuel et al., 2013; Wong and Krainc, 2017). Many recent studies also suggested a “prion-like” cell-to-cell transmission of α-Syn as a critical molecular event underlying the spread of α-Syn pathology and disease progression in PD (Masuda-Suzukake et al., 2013; Brundin and Melki, 2017; Goedert et al., 2017). Currently, there are no successful treatments available that can slow or halt this chronic neurodegeneration (Shoulson, 1998; Mattson, 2004; Olanow, 2004; Lopez-Diego and Weiner, 2008; Mestre et al., 2009).

In the absence of an effective treatment for PD, epidemiological and experimental investigations into potential risk factors (including dietary factors) that may allow individuals to decrease their risk for neurodegenerative disorders has become more appealing. Caffeine is doubtless the most widely consumed psychoactive substance. It is estimated that more than 50% of the world's adult population consume caffeine daily (Fredholm et al., 1999). Since 2000, several large prospective epidemiological studies have shown an inverse relationship between the consumption of caffeinated (but not decaffeinated) coffee and the risk of developing PD, including the Honolulu Heart Program (Ross et al., 2000), the Health Professionals' Follow-Up Study and the Nurses' Health Study (Ascherio et al., 2001), and the Finnish Mobile Clinic Health Examination Survey (Saaksjarvi et al., 2007). A recent meta-analysis of 110 observational studies has firmly established a relationship between increased caffeine consumption and decreased risk of developing PD (Grosso et al., 2017). Despite considerable strengths of epidemiological evidence, the therapeutic potential of caffeine remains unclear. In particular, there are concerns over the failure of recent clinical trials to demonstrate the symptomatic therapeutic benefits in PD patients (Simon et al., 2015; Postuma et al., 2017).

Moreover, studies with animal models of PD provide a compelling clue about the potentially protective effects of caffeine by demonstrating that caffeine treatment attenuates dopaminergic neurotoxicity and neurodegeneration in MPTP model of PD. Importantly, pharmacological blockade or genetic inactivation of adenosine A_2A_ receptor (A_2A_R, the main pharmacological target of caffeine in the brain) attenuates dopaminergic neurotoxicity and neurodegeneration (Chen et al., 2001; Ikeda et al., 2002; Xu et al., 2002), suggesting that the protective effects of caffeine are due to its action on the A_2A_R (Chen, 2014). Given the central role of pathological forms of α-Syn in PD pathogenesis, recent studies show that A_2A_R blockade decreases α-Syn aggregation in SynT-Synphilin-1 neuroglioma cells (Ferreira et al., 2017a) and rescue synaptic and cognitive deficits in aSyn-transgenic mice (Ferreira et al., 2017b) and A_2A_R gene disruption protects in α-Syn model of PD by preventing loss of dopamine and dopaminergic neurons (Kachroo and Schwarzschild, 2012). Moreover, we show that genetic deletion of A_2A_Rs attenuates neurodegeneration and cognitive impairments with reduced microglial and NF-kb activation in α-Syn fibril model of PD (Hu et al., 2016). Recently, caffeine has also been shown to attenuate the toxicity of α-Syn aggregates *in vitro* and in yeast proteotoxicity model by direct binding of caffeine with α-Syn proteins (Kardani and Roy, 2015). However, whether caffeine can modulate mutant α-Syn-induced neurotoxicity in intact animal models of PD has not been examined.

Considering the possible mechanism associated with the postulated effect of caffeine, both caffeine and A_2A_R signaling can regulate autophagy activity under different conditions in several cell types (Sinha et al., 2014; Liu et al., 2016). Furthermore, increasing evidence demonstrates that aberrant regulation of autophagy, as one of the main systems involved in the proteolytic degradation of α-Syn, contributes to the aggregation of α-Syn and α-Syn-induced neurodegeneration in PD (Ebrahimi-Fakhari et al., 2012; Poehler et al., 2014; Xilouri et al., 2016). Thus, we postulated that caffeine protects against α-synucleinopathy by modulating autophagy activity in intact animal models of PD.

In the present study, we implemented the well-established α-Syn fibril model of PD by intra-cerebral injection of preformed A53T α-Syn fibrils to explore the caffeine's protective effect and associated mechanisms. This PD model has been recently shown to recapitulates many features of PD, including neuron loss and robust formation of α-Syn inclusions, which resemble biochemical and morphological features of Lewy bodies and Lewy neurites found in the PD brain (Volpicelli-Daley et al., 2011; Luk et al., 2012a), and the abnormal seeking and spreading of α-Syn inclusions throughout the brain. This is in contrast with α-Syn soluble oligomeric species that are thought to be the most neurotoxic species, but they apparently do not cause inclusion formation (Volpicelli-Daley et al., 2011, 2014). Using this PD model coupled with the chronic caffeine treatment paradigm to resemble human caffeine consumption, we provided the direct evidence that chronic caffeine treatment confers neuroprotective effect by blunting a cascade of pathological events leading to α-Syn pathology, including pSer129α-Syn-rich aggregates, neuroinflammation, and apoptotic neuronal cell death. Furthermore, this protective effect of caffeine is associated with the enhanced activity of autophagy (specifically macroautophagy and chaperone-mediated autophagy, CMA). These findings support that caffeine—a strongly protective environment factor by epidemiological evidence—may represent a novel pharmacological therapy for PD by targeting autophagy pathway.

## Materials and methods

### Animals

All experimental procedures were conducted in accordance with the US National Institutes of Health Guide for the Care and Use of Laboratory Animals and the Animal Experimentation Regulation of Wenzhou Medical University, China. The C57BL/6 male mice (10–12 weeks old, weighting 22–26 g) were provided by Beijing Vital River Laboratory Animal Technology Co., Ltd. and maintained under the controlled environment (23 ± 2°C; 12 h light/dark cycle; *ad libitum* access to food and water).

### Preparation and analysis of recombinant α-Syn fibrils

Purification of recombinant α-Syn proteins and *in vitro* fibril formation was performed as previously described (Luk et al., 2012b) and validated by electron microscopic analysis. Briefly, full-length cDNA of human α-Syn-His6 containing A53T mutation was synthesized and cloned into *E. coli* expression vector pET24a. The expression vector was then used to transform BL21 (DE3) cells. Appropriate chromatography including nickel affinity and gel filtration was implemented to purify the target proteins. Protein concentrations (over 90% purity) were determined using the BCA kit (Beyotime Biotechnology) with BSA as a standard. The nature of α-Syn fibrils was assessed using a H-7650 (Hitachi Ltd, Japan) transmission electron microscope (TEM) after adsorption of the samples onto carbon-coated 200-mesh grids and negative staining with 1% uranyl acetate. The images were acquired with a GatanOrius CCD camera (Gatan, US).

### Intra-striatal injection of preformed A53T α-Syn fibrils

The injection of α-Syn fibrils was performed as previously described (Luk et al., 2012a,b; Hu et al., 2016). The α-Syn fibrils were incubated at 37°C at 5 mg/ml in sterile PBS with shaking at 1,000 rpm and diluted to a concentration of 1.54 mg/ml in sterile PBS, then α-Syn fibrils were briefly sonicated before intracerebral injection. For histological analysis of α-Syn-induced pathology, male mice with 10–12 weeks old (weighting 22–26 g) were bilaterally injected with 3.25 μl (5 μg) A53T α-Syn or 3.25 μl sterile PBS into the striatum (coordinates from bregma: AP, +0.98 mm; ML, ±2.25 mm; DV, −2.6 mm). The injection was performed stereotaxically at a rate of 0.15 μl/ min (total volume of 3.25 μl/ site) with the needle in place for 10 min before its withdrawal.

### Chronic caffeine treatment

The caffeine chronic treatment was adapted from our previous studies of caffeine-mediated protection against traumatic brain injury (Li et al., 2008; Chen et al., 2010). Caffeine (Sigma Aldrich, C0750) solution was freshly prepared at concentration of 1 g/L in drinking water and was given to mice (10–12 weeks old). This dosage produced plasma caffeine and metabolite levels at the concentration of ~0.4–2 mg/L, comparable to those obtained by regular human coffee consumption (~1–4 mg/L) (Fredholm et al., 1999). Indeed, we measured the blood levels of caffeine in this study by HPLC and confirmed that the blood levels of caffeine are 5.271 ± 2.457 mg/L. Caffeine treatment was initiated 7 days before the mice received intra-striatal injections of PBS or A53T α-Syn fibrils and continuously lasted for 120 days. The mice exposed to water only served as a control of vehicle treatment.

### Immunohistochemical and immunofluorescence analyses

Mice were deeply anesthetized using 3.6% chloral hydrate with an overdose and then transcardially perfused with saline and 4% paraformaldehyde. Brains were removed and post-fixed in 4% paraformaldehyde for 4–6 h at 4°C, and then equilibrated using gradient sucrose solution (10, 20, and 30%). Brain sections (30 μm) were cut with a freezing microtome (Leica CM 1950). Immunohistochemistry and immunofluorescence were performed on 30 μm free-floating sections. Free-floating sections were washed in 0.01M PBS (pH = 7.4) and then incubated for 60 min in 0.3% TritonX-100 and 10% normal donkey serum. Primary antibodies were incubated following manufacturers' protocols: pSyn (Wako, 1:1,000, 015-25191), GFAP (Sigma-Aldrich, 1:400, G3893), Iba-1 (WAKO, 1:200, 019-19741), LC3B (Abcam, 1:200, ab48394), SQSTM1/p62 (Abcam, 1:100, ab56416), LAMP2A (Abcam, 1:200, ab18528). For immunofluorescence analysis, brain sections were incubated with Alexa 488-conjugated secondary antibodies (Invitrogen, A-11029, 1:1,000) or Alexa 594-conjugated secondary antibodies (Invitrogen, A-11007, 1:1,000). The sections were washed and mounted. Fluorescent mounting medium (contain DAPI) were applied on the sections. Images were acquired by a fluorescence microscope and quantified as MOD (mean optical density) using Image Pro Plus. Fluorescent double staining was visualized by confocal microscopy (LSM710, Zeiss). For immunohistochemical analysis, the sections were incubated with the Biotin-SP-AffiniPure Donkey Anti-Mouse IgG (H+L) (Jackson immuno-research, 1:1,000, 715-065-151) or Biotin-SP-AffiniPure Fab Fragment Donkey Anti-Rabbit IgG (H+L) (Jackson immuno-research, 1:1,000, 711-067-003). The sections were then immunostained using the avidin-biotin complex (ABC) system (VECTASTAIN Elite ABC-HRP Kit, Vector Laboratories, PK-6100) and immunocomplexes were visualized by DAB Peroxidase (HRP) Substrate Kit (Vector Laboratories, SK-4100). The sections were counterstained with hematoxylin and images were acquired with a bright-field microscope.

### TUNEL staining for detection of cell apoptosis

The TdT-mediated dUTP Nick-End Labeling (TUNEL) staining was performed on 30-μm sticking frozen brain sections using a commercial In Situ Cell Death Detection Kit (Roche Diagnostics, Basel, Switzerland). Briefly, striatal sections (30 μm) were permeabilized and antigen retrieval was performed by 0.1% sodium citrate buffer solution with 0.1% Triton X-100 for 5 min at 4°C. After washing for 3 times, the sections were incubated in TUNEL reaction solutions for 1 h at 37°C, and then washed. Fluorescent mounting medium (contain DAPI) was applied onto the sections. Fluorescent double staining was visualized by confocal microscopy (LSM710, Zeiss).

### Western blot analysis

The mice were anesthetized using 3.6% chloral hydrate and then decapitated. Striatal tissues from these bilaterally injected mice were dissected out and processed for Western blot analysis. Briefly, after measurement of protein concentration, samples were diluted with five volumes of SDS-PAGE buffer. These diluted samples were separated by SDS-PAGE (12% with a 5% concentrating gel) in reducing conditions and electro-transferred to PVDF membranes (Millipore, US). After blocking for 2 h at room temperature with 5% milk in Tris-buffered saline (pH = 7.6) containing 0.05% Tween-20 (TBS-T), the membranes were incubated overnight at 4°C with anti-LC3B antibody (Abcam, 1:1,000, ab48394), anti-Beclin1 antibody (Abcam, 1:500, ab62557), anti-SQSTM1 antibody (Abcam,1:1,000, ab56416), anti-LAMP2A antibody (Abcam, ab18528,1:1,000), anti-Hsc70 antibody (Abcam,1:1,000, ab2788), anti-PSMC3 (Abcam, ab171974, 1:1,000), anti-Proteasome 20S beta 6 (abcam, ab150392, 1:1,000) and β-actin (Abcam, ab8226, 1:1,000). After three washes, the membranes were incubated with goat anti-Rabbit secondary antibody (Li-COR, P/N 925-32211, 1:5,000) or goat anti-Mouse secondary antibody (Li-COR, P/N 925-32210, 1:5,000) for 2 h at room temperature. The images of Western blot were taken by LI-COR Odyssey.

### RNA isolation and quantitative PCR

The mice were anesthetized using 3.6% chloral hydrate and then decapitated. Striatal tissues from these bilaterally injected mice were processed for quantitative PCR analyses. Striatal tissues were homogenized in TRIzol reagent (Life Technologies, 15596-026), and total RNA was extracted according to the manufacturer instructions. Concentration and purity of total RNA were then determined using NanoDrop ND 1,000 (Thermo Scientifics). First cDNA chain was synthesized from 1 μg of extracted total RNA using PrimeScript™ RT Master Mix (Perfect Real Time) (Takara) according to the manufacturer's protocol. Quantitative PCR was performed with SYBR-Green premix Extaq (Takara) and detected by a Real Time PCR System (CFX96; Bio-Rad). GAPDH was used as an internal control gene. The following primers were used for *map1lc3b* (forward: 5′-ACAAAGAGTGGAAGATGTCCG-3′ and reverse: 5′-CCCCTTGTATCGCTCTATAATCAC-3′), *beclin1* (forward: 5′-AGGAACTCACAGCTCC ATTAC-3′ and reverse: 5′-AATGGCTCCTCTCC TGAGTT-3′), *sqstm1* (forward: 5′-CCTATA CCCACATCTCCCACC-3′ and reverse: 5′-TGTCG TAATTCTTGGTCTGTAGG-3′), *lamp2a* (forward: 5′-TGTATTTGGCTAATGGCTCAGC-3′ and reverse: 5′-TATGGGCACAAGGA AGTTGTC-3′) and *hsc70* (forward: 5′-TCTCGGCACC ACCTACTCC-3′ and reverse: 5′- CTACGCCCGATCAGACGTTT-3′). A standard two-step PCR amplification was performed, with a melting step at 95°C for 15 sand annealing and elongation at 60°C for 30 s, for 40 cycles. Following PCR amplification, a first derivative melting-curve analysis was conducted to confirm the specificity of the PCR. Ct values were converted to relative quantification using the 2^−ΔΔCt^ method.

### Statistical analysis

All quantitative assessments of immunohistochemistry and immunofluorescence staining results were made by an investigator who was blind to the treatments of the experimental animals. For quantitative analysis, three sections per mouse were selected and cell counts for each section were obtained. These cell counts were normalized to the total number of cells as labeled by DAPI. Cell counts from three sections were averaged for each mouse. Immunostaining and western blotting images were all quantified by Image J software. All statistical analyses were carried out using SPSS Statistics 19.0 (SPSS Software) and GraphPad Prism 7.0 (GraphPad Software). The data were presented as mean ± SEM. All experimental data were analyzed by two-way ANOVA (with a-Syn and caffeine treatments as two factors), followed by Bonferroni *post-hoc* test for multiple experimental group comparisons. *p* < 0.05 was considered statistically significant.

## Results

### Chronic caffeine treatment attenuates p129-positive α-Syn inclusions induced by intra-striatal injection of A53T α-Syn fibrils

**Figure 1:**
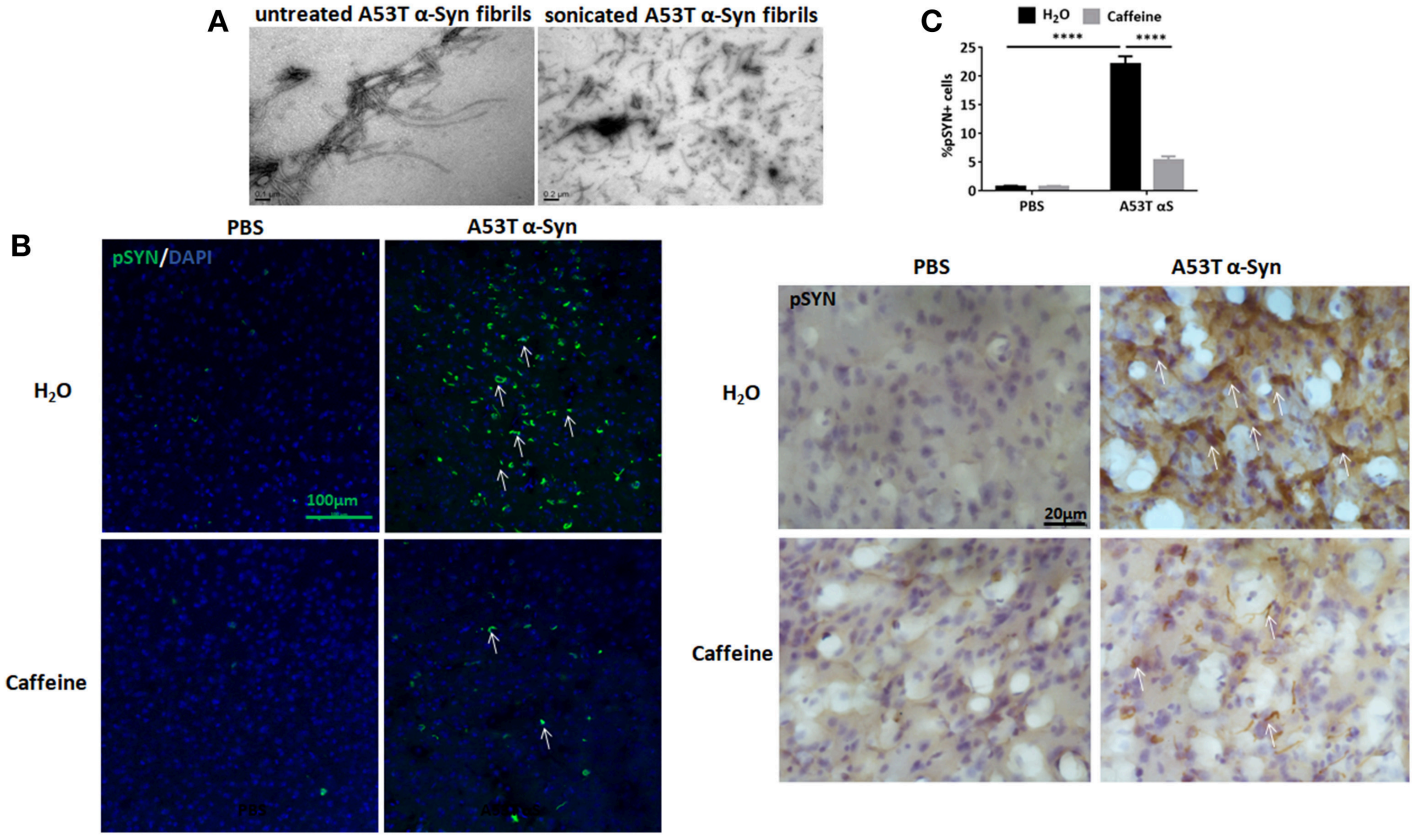
Chronic caffeine treatment attenuates p129-positive α-Syn inclusion induced by A53T α-Syn fibrils. C57B/L6 mice were bilaterally injected with PBS or A53T α-Syn fibrils into the striatum and treated with caffeine or drinking. Mice striatum were analyzed for α-Syn inclusions 4 months after the injection and treatment. **(A)** Electron micrographs of preformed A53T α-Syn fibrils before and after sonication. **(B)** Immunofluorescence and immunohistochemistry staining of p129-positive α-Syn inclusions in the striatum were mainly detected in the mice injected with A53T α-Syn fibrils. The p129-Syn-inclusions were attenuated by chronic caffeine treatment at 4 months. **(C)** Quantitative analysis of cells containing p129-Syn-positive inclusions. After the injection of A53T α-Syn fibrils, the number of p129-Syn-positive cells was significantly increased in striatum (*n* = 4/group, ^****^p < 0.0001). Following chronic caffeine treatment, the signal of p129-Syn-positive cells was decreased markedly (*n* = 4/group, ^****^p < 0.0001). Scale bar: 100 and 20 μm.

To explore the neuroprotection against synucleinopathy by caffeine, we used the intra-striatal injection of full-length human A53T α-Syn fibrils to induce synucleinopathy as we described previously (Hu et al., 2016). Figure 1A shows the preformed A53T α-Syn fibrils by electron microscopy analysis. Caffeine treatment via drinking water was given 7 days prior to the α-Syn injection and lasted for about 4 months. At about 120 days after the injection, α-Syn inclusion pathology with typical morphology of Lewy bodies or Lewy neurites was detected at and near the injection site in the striatum (Figure 1B). Consistent with our previous study, whereas the mice injected with PBS did not exhibit α-Syn inclusion, the mice injected A53T α-Syn fibrils displayed prominent α-Syn inclusion pathology, as evident by the presence of p129-positive α-Syn aggregates around the striatal injection site (Figure 1B). Percentage of striatal cells with α-Syn inclusion was increased compared to the PBS control group (Figure 1C, *n* = 4/group, ^****^p < 0.0001). Importantly, chronic caffeine treatment markedly attenuated the α-Syn inclusion pathology, with reduced and diffused staining of p129-positive α-Syn inclusion in the striatum. Percentage of cells with α-Syn inclusion was significantly decreased in A53T group treated with caffeine compared to the A53T-water treatment group (Figure 1C, *n* = 4/group, ^****^p < 0.0001).

### Chronic caffeine treatment attenuates A53T α-Syn-induced cellular apoptosis in the striatum

**Figure 2:**
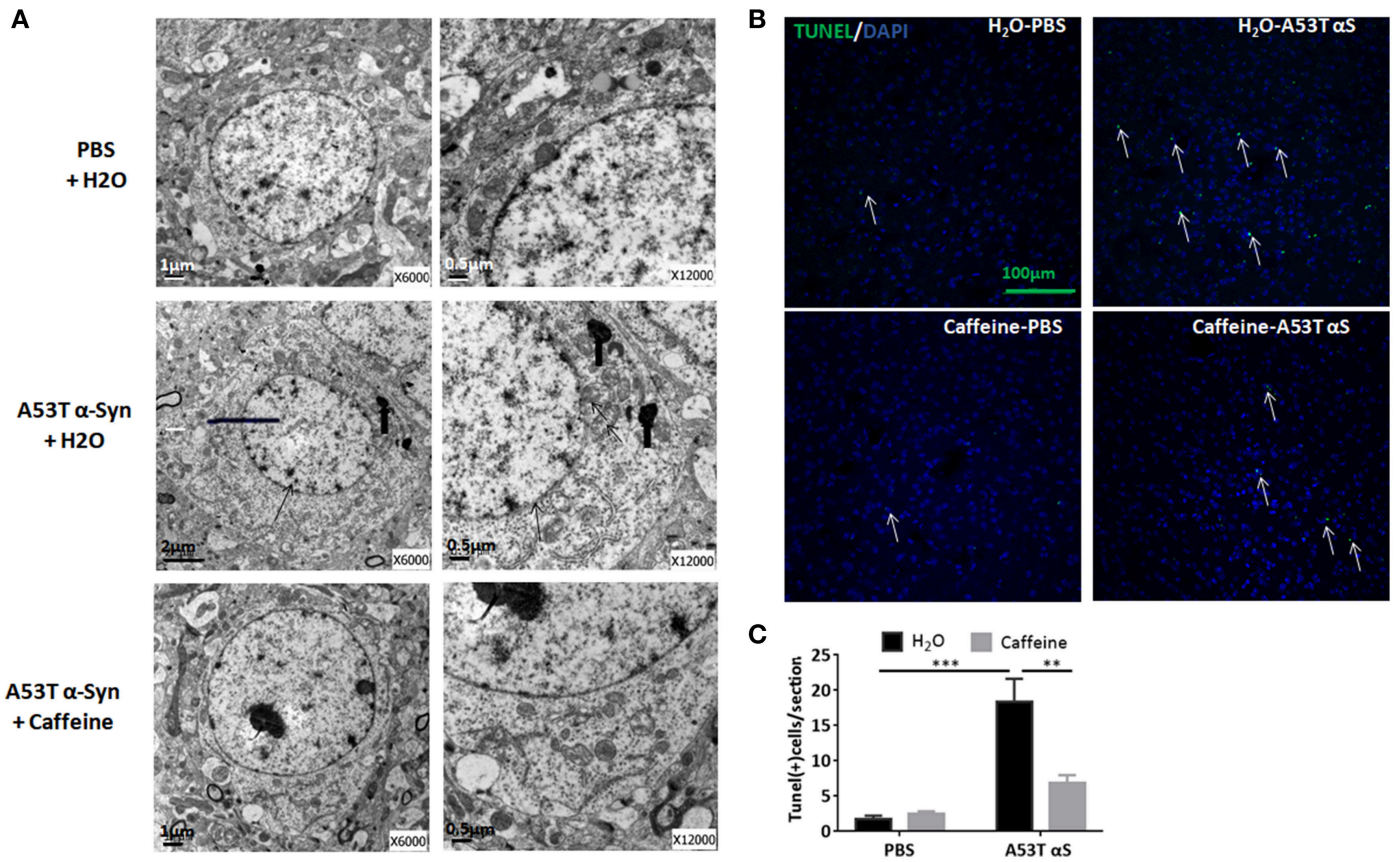
Chronic caffeine treatment attenuates A53T α-Syn-induced cellular apoptosis in the striatum. C57B/L6 mice were bilaterally injected with PBS or A53T α-Syn fibrils into the striatum and treated with caffeine or water. Mice striatum were analyzed for apoptosis 4 months after the injection and treatment. **(A)** Electronic microscope analysis of cellular apoptosis. The thin arrows indicate A53T α-Syn fibrils-induced fuzzy boundary of the nuclear membrane; thick arrows point to accumulation of lipofuscin; arrowheads point to the cristae fractured of swelling mitochondria. **(B)** The TUNEL-positive apoptotic cells (green) were detected in the striatal sections of the mice injected with PBS or A53T α-Syn fibrils and treated with water or caffeine. Cellular nuclei were stained with DAPI (blue). **(C)** Quantitative analysis of TUNEL-positive cells per section of each group (*n* = 4/group, ^***^p = 0.0001, ^**^p = 0.0036). Scale bar: 100 μm.

To explore neuroprotection by caffeine against α-synucleinopathy, we examined α-Syn-triggered neuronal death by electron microscopy analysis and by TUNEL assay in the striatum of caffeine-treated and water-treated mice. As shown in Figure 2A, after the striatal injection, A53T α-Syn fibrils triggered a neuronal cell death pathology characterized by decreased size of cells, fuzzy boundary of the nuclear membrane, increased heterochromatin, accumulation of lipofuscin and cristae fractured of swelling mitochondria at the ultra-structural level. Importantly, chronic caffeine treatment apparently reverted these ultrastructural changes. Similarly, at the cellular level, prominent apoptosis was induced by A53T α-Syn fibrils compared with the PBS-treated control mice (Figure 2B). The number of TUNEL-positive cells induced by A53T α-Syn fibrils was elevated compared to the PBS control (Figure 2C, *n* = 4/group, ^***^p = 0.0001). Notably, chronic caffeine treatment reduced the number of TUNEL-positive cells induced by α-Syn (Figure 2C, *n* = 4/group, ^**^p = 0.0036). Thus, caffeine protected against synucleinopathy by modulating α-Syn-induced apoptosis in the striatum.

### Caffeine attenuates A53T α-Syn-induced microglial activation and astrogliosis in the striatum

**Figure 3:**
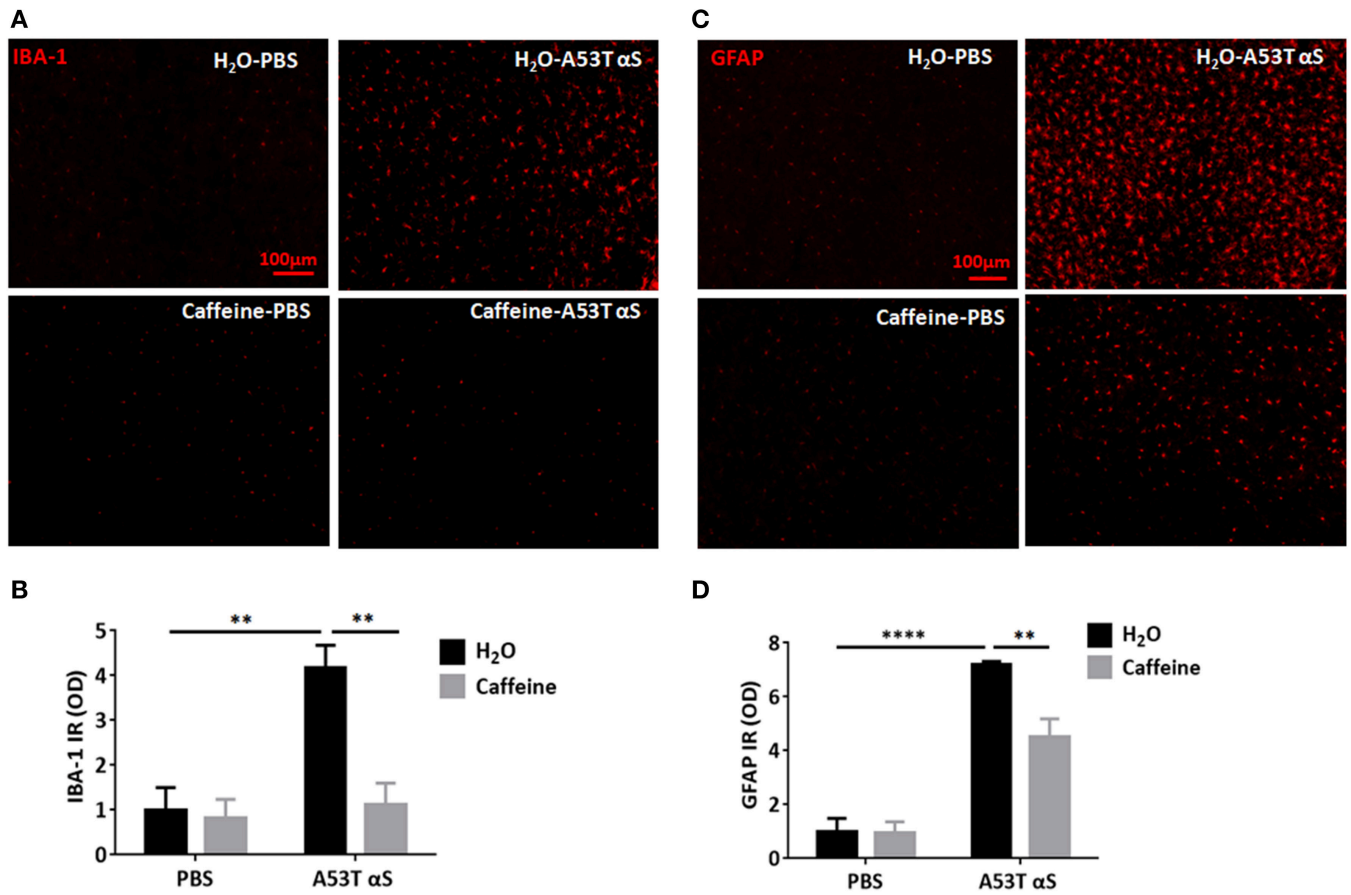
Chronic caffeine treatment attenuates A53T α-Syn-induced microglial activation and astrogliosis in the striatum. C57B/L6 mice were bilaterally injected with PBS or A53T α-Syn fibrils into the striatum and treated with caffeine or water for 4 months. Mice striatum were analyzed for IBA1-positive microglial activation and GFPA-positive reactive astrogliosis 4 months after surgery and treatment. **(A,C)** A53T α-Syn fibrils triggered marked microglial activation with IBA1-positive cells (red) and astrogliosis with GFAP immunoreactivity (red) in the striatum. **(B,D)** Quantitative analysis of the mean optical density of IBA-1 and GFAP immunoreactivity. IBA1-positive and GFAP-positive cells in group injected with A53T α-Syn fibrils increased by 4.16 and 7.2-folds, respectively, comparing to PBS control (*n* = 4/group, ^**^p = 0.0029, ^****^p < 0.0001). Chronic caffeine treatment significantly reduced the α-Syn-induced microglial activation and reactive astrogliosis (*n* = 4/group, ^**^p = 0.0042, ^**^p = 0.0095). Scale bar: 100 μm.

As α-Syn-induced neuroinflammation is crucial to consequent neuronal death, we further investigated whether caffeine prevented synucleinopathy by microglial activation and astrogliosis during neuroinflammation. We examined both ionized calcium binding adaptor molecule-1 (IBA1)-positive microglial activation and glial fibrillary acidic protein (GFPA)-positive reactive astrogliosis after the intra-striatal injection of A53T α-Syn fibrils. As shown in Figures 3A,C, A53T α-Syn fibrils triggered marked microglial activation with IBA1-positive cells and astrogliosis with GFAP immunoreactivity in the striatum. Quantitative analysis of the mean optical density of IBA-1 and GFAP immunoreactivity showed that IBA1-positive and GFAP-positive cells increased by 4.16- and 7.2-folds in comparison with the PBS control, respectively (Figures 3B,D, *n* = 4/group, ^**^p = 0.0029, ^****^p < 0.0001). Importantly, chronic caffeine treatment largely reverted the α-Syn-induced microglial activation and reactive astrogliosis (compared to the water-treated, A53T α-Syn fibrils-injected mice), respectively (Figures 3B,D, *n* = 4/group, ^**^p = 0.0042, ^**^p = 0.0095). Thus, caffeine may confer neuroprotection against synucleinopathy by modulating neuroinflammation (microglial and astrocytic activation) in the striatum.

### Chronic caffeine treatment does not affect ups activity but selectively reverses the defects in macroautophagy and CMA induced by A53T α-Syn fibrils in the striatum

Removal of protein aggregates such as misfolded mutant A53T α-Syn fibrils can occur via the autophagy-lysosomal pathway and ubiquitin proteasome system (UPS). To explore this possible involvement of UPS in α-Syn fibrils model of PD we examined the effect of caffeine on the level of PSMC3 and proteasome 20S beta 6 protein, as indicator of UPS activity because both are mainly degraded by UPS system. We found that neither α-Syn nor caffeine treatment affect the levels of PSMC3 and proteasome 20S β6 protein in the striatum, indicating the lack of UPS system in our experiment model (see Supplementary Figure 1). Considering this preliminary analysis coupled with previous findings of activation of UPS by A_2A_R agonists (Chiang et al., 2009) (not antagonist such as caffeine) and preferentially clearance of aggregated α-Syn by autophagy (Webb et al., 2003), we have mainly focused on the analysis of autophagy pathway in our study.

**Figure 4:**
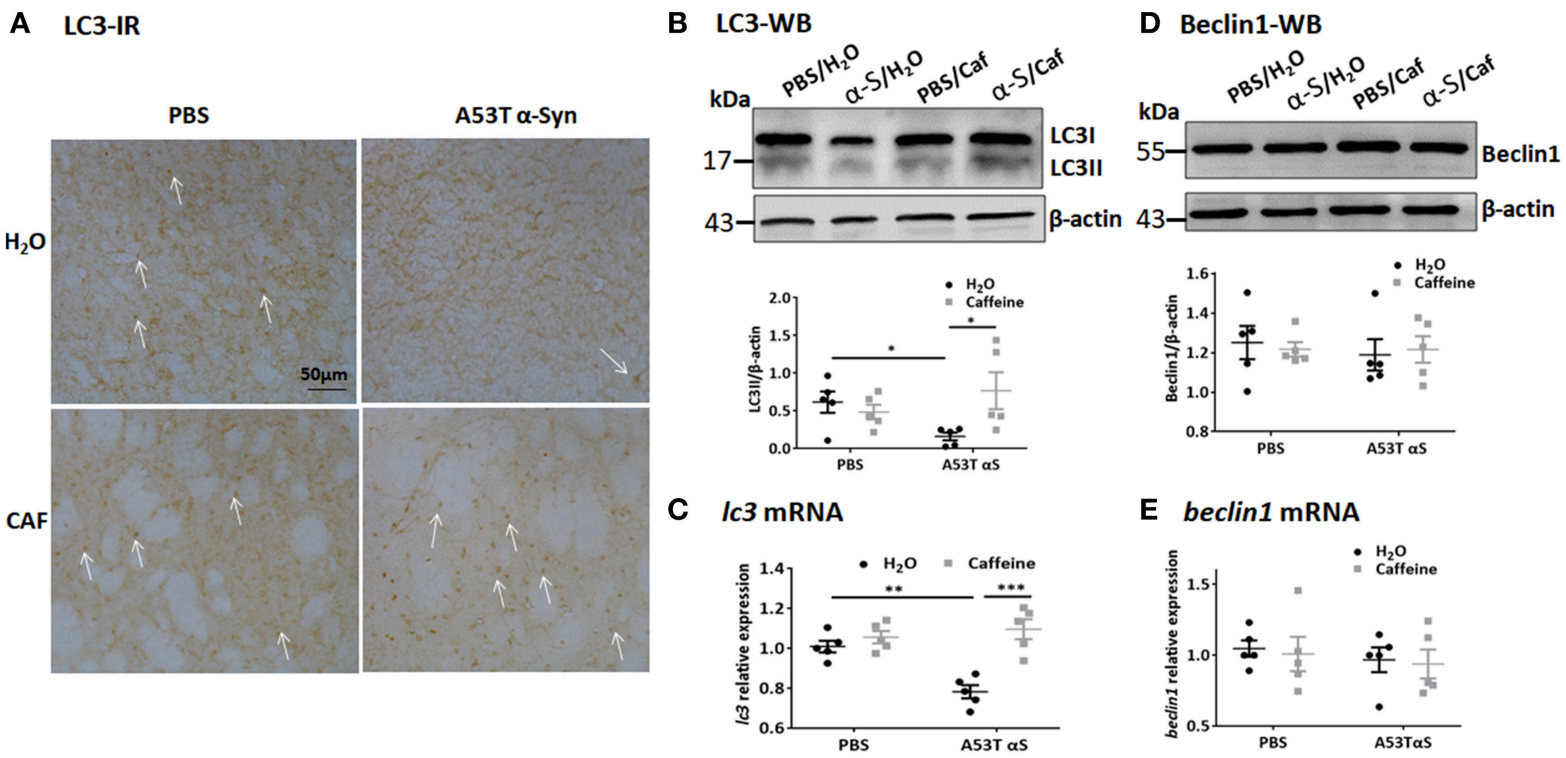
Chronic caffeine treatment reverses LC3 defect induced by A53T α-Syn fibrils C57B/L6 mice were bilaterally injected with PBS or A53T α-Syn fibrils into the striatum and treated with caffeine or water. Mice striatum were analyzed for autophagy-related protein LC3 and beclin1 by Western blot and immunohistochemistry 4 months after the injection and treatment. **(A)** Immunohistochemistry of LC3 in sections of striatum. Injection of A53T α-Syn fibrils markedly reduced LC3-immunoreactivity in the striatum. Chronic caffeine treatment completely reversed these defects in LC3-immunoreactivity. Arrows indicate the positive signals of LC3. **(B,D)** Western blot analysis of LC3 and Beclin1 from the striatal tissue lysates. Injection of A53T α-Syn fibrils reduced LC3II level compared to control group (*n* = 5/group, ^*^p = 0.0497). Chronic caffeine treatment reversed the reduction of LC3-II induced by A53T α-Syn fibrils (*n* = 5/group, ^*^p = 0.0121). **(C,E)** QPCR analysis of *lc3* and *beclin1*. Injection of A53T α-Syn decreased the *lc3* mRNA level (*n* = 5/group, ^**^p = 0.003), chronic caffeine treatment reversed the reduction of *lc3* mRNA level induced by A53T α-Syn fibrils (*n* = 5/group, ^***^p = 0.0001). The expression levels of *beclin1* were not altered (*n* = 5/group*, p* > 0.05). Scale bar: 100 μm.

The autophagy-lysosomal pathway is prominently mediated by macroautophagy and chaperone-mediated autophagy (CMA). Macroautophagy involves the formation, elongation and nucleation of autophagosomes with double-membrane organelles to sequester the substrate before fusion with lysosomes. We exploited the immunohistochemical and Western blot analyses of LC3 (microtubule-associated protein 1 light chain 3) aggregates (Figures 4A,B) was used to detect the generation of autophagosomes, with the LC3 antibody recognizing two LC3 proteins (distinguished by Western blot analysis): cytosol-associated LC3-I and the autophagosome membranes-associated LC3-II. LC3-immunoreactivity with puncta morphology was detected in the striatal cell bodies. Notably, injection of A53T α-Syn fibrils markedly reduced LC3-immunoreactivity in the striatum (Figure 4A). Chronic caffeine treatment not only completely reversed these defects in LC3-immunoreactivity, but apparently increased LC3-immunoreactivity above the normal level compared to the control (PBS-water) group (Figure 4A). Western blot analysis confirmed the immunohistochemistry results by quantitatively showing the reduction of LC3-II in A53T α-Syn-treated striatum (Figure 4B, *n* = 5/group, ^*^p = 0.0497). Chronic caffeine treatment reversed α-Syn-induced decrease in LC3-II level compared to the A53T α-Syn-water treatment group (Figure 4B, *n* = 5/group, ^*^p = 0.0121). However, analysis of LC3-II/LC3-I ratio only showed a similar trend of reduced LC3-II/LC3-I ratio by a-Syn and reversal of this reduction by caffeine. Furthermore, qPCR analysis validated this finding by showing that A53T α-Syn injection reduced the *lc3* mRNA level in the striatum (Figure 4C, *n* = 5/group, ^**^p = 0.003). Chronic caffeine treatment did not exert any effect on normal striatum, but reversed α-Syn-induced decrease in the *lc3* mRNA (Figure 4D, *n* = 5/group, ^***^p = 0.0001), suggesting that caffeine modulated LC3-mediated macroautophagy at least partially by a transcription mechanism.

**Figure 5:**
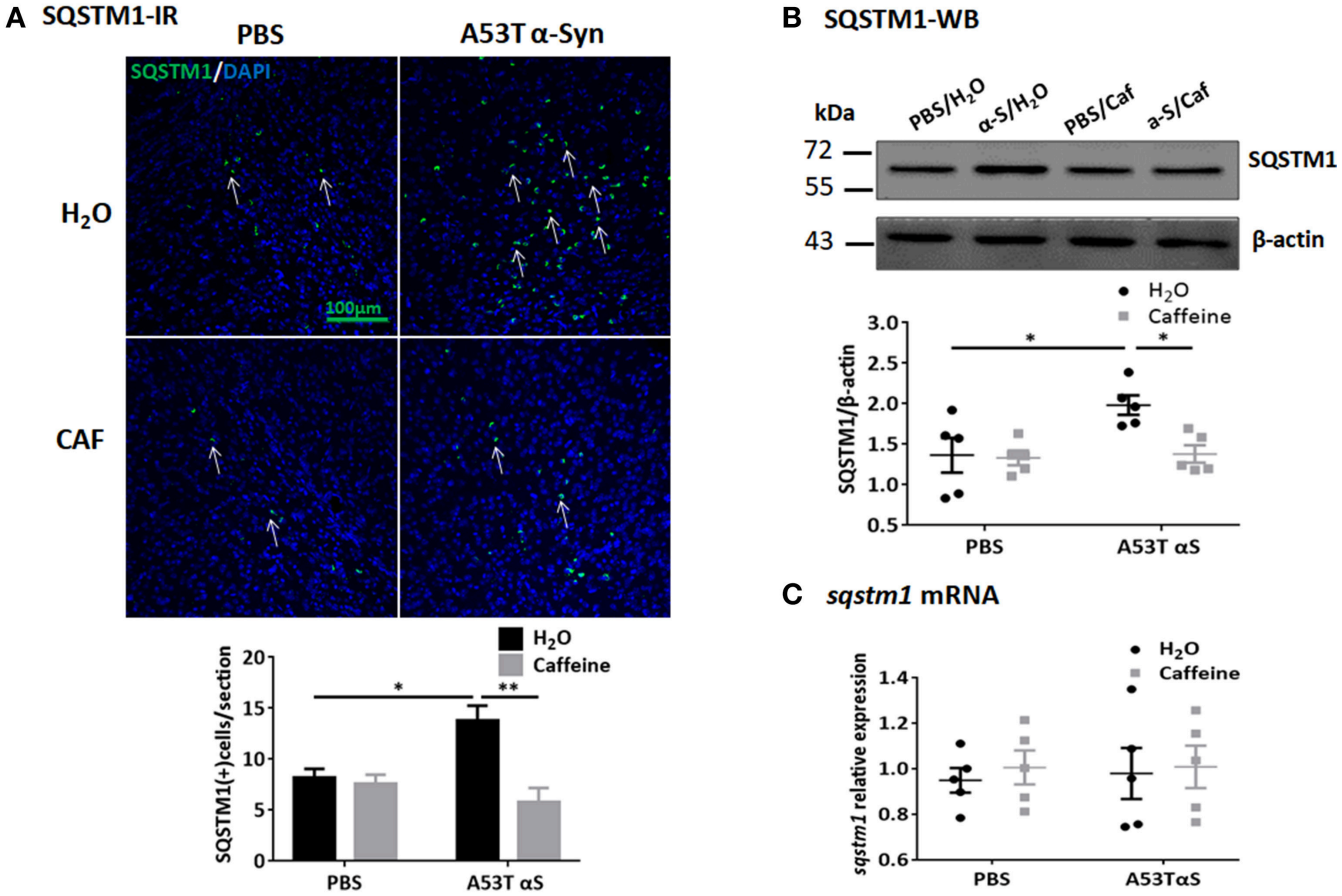
Chronic caffeine treatment attenuates SQSTM1 induction by A53T α-Syn fibrils. C57B/L6 mice were bilaterally injected with PBS or A53T α-Syn fibrils into striatum and analyzed for SQSTM1 4 months after surgery and treatment. **(A)** Immunofluorescence staining of SQSTM1 (green) in sections of striatum. Injection of A53T α-Syn fibrils markedly induced SQSTM1-immunoreactivity in the striatum, whereas chronic treatment with caffeine attenuated this induction (*n* = 4/group, ^*^p = 0.0378, ^**^p = 0.0027). Cellular nuclei were stained with DAPI (blue). **(B)** Western blot analysis of SQSTM1 from the striatal tissue lysates.Injection of A53T α-Syn fibrils increased the level of SQSTM1 protein compared to control group (*n* = 5/group, ^*^p = 0.0423), and chronic caffeine treatment attenuated the induction of SQSTM1 by A53T α-Syn (*n* = 5/group, ^*^p = 0.0498). **(C)** QPCR analyses of *sqstm1*. The expression patterns of *sqstm1* were not altered (*n* = 5/group, *p* > 0.05). Scale bar: 100 μm.

To further study the dynamic process of autophagy, we examined the levels of macroautophagy-related protein Beclin1 (a marker for the initiation of macroautophagic flux), and the receptor protein sequestome 1 (SQSTM1/p62; a protein involved in delivery of ubiquitinated cargo to autophagosomes). We found that the protein and mRNA levels of Beclin1 were not affected by either A53T α-Syn or caffeine treatment as revealed by Western blot and qPCR analyses (Figures 4D,E, *n* = 5/group, *p* > 0.05). Injection of the α-Syn fibrials into the striatum increased the immunoreactivity and the level of SQSTM1 protein compared to the control (PBS-treated) group (Figure 5A, *n* = 4/group, ^*^p = 0.0378), whereas chronic caffeine treatment decreased the SQSTM1 protein levels as detected by immunofluorescence and Western blot analyses (Figure 5A, *n* = 4/group, ^*^p = 0.0027, Figure 5B, *n* = 5/group, ^*^p = 0.0498). The expression patterns of sqstm1 were not altered (Figure 5C, *n* = 5/group, *p* > 0.05).

### Chronic caffeine treatment selectively reverses the defects in CMA induced by A53T α-Syn fibrils in the striatum

**Figure 6:**
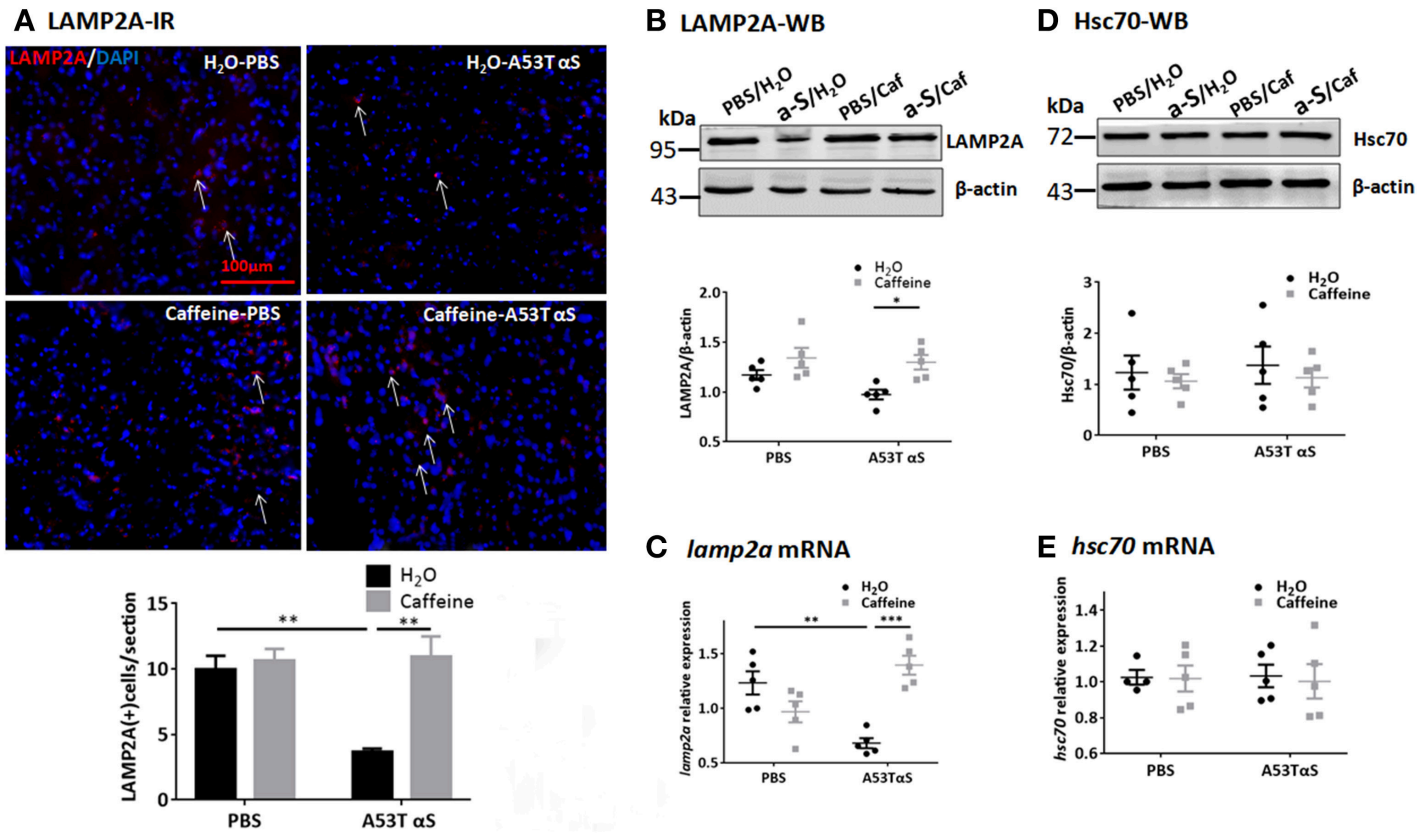
Chronic caffeine treatment reverts level of CMA related protein LAMP2A. C57B/L6 mice were bilaterally injected with PBS or A53T α-Syn fibrils into the striatum and treated with caffeine or water for 4 months. Mice striatum were analyzed for LAMP2A 4 months after surgery and treatment. **(A)** Immunofluorescence staining of LAMP2A (red) in sections of striatum. A53T α-Syn injection reduced the immunoreactivity of LAMP2A compared to PBS control (*n* = 4/group, ^**^p = 0.0073). Chronic caffeine treatment reversed immunoreactivity of LAMP2A (*n* = 4/group, ^**^p = 0.0023). Cellular nuclei were stained with DAPI (blue). Arrows indicate the positive signals of LAMP2A. **(B,D)** Western Blot analysis of LAMP2A and Hsc70. A53T α-Syn injection apparently reduced the LAMP2A protein, whereas chronic caffeine treatment enhanced the LAMP2A protein levels above the control level as detected by Western blot analysis (*n* = 5/group, ^*^p = 0.0313). **(C,E)** QPCR analysis of *lamp2a* mRNA in the striatum. A53T α-Syn injection reduced the *lamp2a* mRNA level (*n* = 5/group, ^**^p = 0.0023), whilst chronic caffeine treatment reversed this reduction (*n* = 5/group, ^***^p = 0.0002) but did not have any effect on normal striatum. The expression levels of *hsc70* were not altered (*n* = 5/group*, p* > 0.05). Scale bar: 100 μm.

We also examined the effect of chronic caffeine treatment on CMA, in which chaperone-complex involving the heat-shock cognate recognizes soluble cytosolic proteins containing a KFERQ-related targeting motif since A53T, A30P α-Syn, and dopamine-modified α-Syn have been shown to blocks CMA and contribute to dopaminergic vulnerability in PD (Xilouri et al., 2016). Western blot and qPCR analysis showed that the Hsc70 protein and *hsc70* mRNA levels in the striatum were not affected by A53T α-Syn injection or chronic caffeine treatment or their co-treatment. However, A53T α-Syn injection reduced the immunoreactivity of LAMP2A (Figure 6A, *n* = 4/group, ^*^p = 0.0073). Chronic caffeine treatment enhanced the LAMP2A protein levels as detected by immunofluorescence (Figure 6A, *n* = 4/group, ^**^p = 0.0023) and Western blot analysis (Figure 6B, *n* = 5/group, ^*^p = 0.0313). Furthermore, qPCR analysis validated the results by showing that A53T α-Syn injection reduced the mRNA level for *lamp2a* in the striatum (Figure 6C, *n* = 5/group, ^**^p = 0.0023) whereas chronic caffeine treatment did not exert any effect on normal striatum, but reversed α-Syn-induced decrease in the *lamp2a* mRNA (Figure 6C, *n* = 5/group, ^***^p = 0.0002). The protein and mRNA levels of Hsc70 were not affected by either A53T α-Syn or caffeine treatment as revealed by Western blot and qPCR analyses (Figures 6D,E, *n* = 5/group, *p* > 0.05).

Collectively, these results suggest that A53T α-Syn fibrils produce defects in macroautophagy and CMA. Chronic caffeine treatment does not affect autophagy processes in the normal striatum, but selectively reverse α-Syn-induced defects in macroautophagy (by enhancing LC3-II and reducing SQSTM1/p62) and CMA (by enhancing LAMP2A).

## Discussion

### Caffeine protects against α-Syn-induced neurotoxicity in A53T α-Syn fibril model of PD

It is critically important to demonstrate that chronic consumption of caffeine can prevent and counteract α-Syn-induced pathology, since current understanding of PD pathogenesis emphasize the central role of pathological forms of α-Syn in PD pathogenesis. This view is based on three genetic and biochemical findings: autosomal dominant mutations in α-Syn in familiar PD, duplications/triplications of *SNCA* gene in some sporadic PD and α-Syn as the major component of Lewy bodies and Lewy neurites (Lashuel et al., 2013). This study used a α-Syn transmission model of PD by intra-striatal injection of preformed mutant (A53T) α-Syn fibrils that produces neuron loss and more importantly robust formation of α-Syn inclusions, which resemble biochemical and morphological features of Lewy bodies and Lewy neurites found in the PD brain (Volpicelli-Daley et al., 2011; Luk et al., 2012a). Moreover, the fibrils are taken up by neurons and induce normal endogenous α-Syn to adopt an abnormal conformation such that it forms inclusions and grows and propagates throughout the brain. This is in contrast with α-Syn soluble oligomeric species that are probably the most neurotoxic species, but they apparently do not cause inclusion formation (Volpicelli-Daley et al., 2011, 2014). Furthermore, we employed a chronic caffeine treatment paradigm via caffeine in drinking water (1 g/L) to achieve plasma concentration (5.271 ± 2.457 mg/L) comparable to the concentration achieved by regular human consumption (≈1–4 mg/L) (Fredholm et al., 1999). Using this α-Syn transmission model of PD coupled with the chronic caffeine treatment paradigm modeling human caffeine consumption, we provide the direct evidence that caffeine protects against α-Syn-induced pathological changes, including α-Syn aggregates (as evident by p129-positive hyper-phosphorylated α-Syn), neuroinflammation (as evident by microglial and astrocytic activation), and cellular apoptosis (TUNEL-positive cells and ultra-structural changes associated cell death) in the striatum.

This conclusion collaborates with three lines of previous investigations in strong support of caffeine-based protection against PD pathology: (i) In a yeast proteotoxicity model with aggregation of recombinant α-Syn, caffeine can reduce the toxicity of oligomers and aggregates, and increase cell survival with concomitant reduction in intracellular oxidative stress and decreased oxidative proteome damage (Kardani and Roy, 2015). This was achieved presumably by caffeine directly binding to α-Syn to alter the nature and confirmation of mature α-Syn aggregates displaying amorphous as well as fibrillar morphology (Kardani and Roy, 2015). (ii) As the A_2A_R is the main pharmacological target of caffeine at the concentration attainable by regular caffeine consumption, our finding also collaborates with the transgenic studies that genetic knockout or pharmacological blockade of the A_2A_R protect α-Syn-induced dopaminergic neurotoxicity and related pathological changes in cultured cells and intact animals (Kachroo and Schwarzschild, 2012; Hu et al., 2016; Ferreira et al., 2017a) and with the recent finding that A2AR antagonists protect against a-Syn-induced synaptic and cognitive deficits with normalized NMDAR2B and PrPC levels in Thy1-a-Syn mice (Ferreira et al., 2017b). These findings collectively provide the strong neurobiological basis for the inverse relationship between coffee consumption and PD risk and support caffeine treatment as a novel prophylactic strategy to alleviate the PD pathology.

This demonstration constitutes a new option for the control of PD pathogenesis by caffeine-mediated autophagy activity with multiple benefits and several favorable features. First, caffeine has unparalleled safety profiles as it is regularly and widely consumed by >50% of the world's adult population. Second, preclinical studies have suggested multiple benefits of caffeine in PD, including motor enhancement, potential neuroprotective effect and cognitive improvement in PD (Chen et al., 2013), although the recent clinical trials have not produced evidence in supporting for clinical use of caffeine in PD (Simon et al., 2015; Postuma et al., 2017). Third, we can identify a more homogenous patient population with sensitivity to caffeine consumption by genetic screening of DNA polymorphisms of genes associated with caffeine consumption, before trial enrollment.

Importantly, the corruption of endogenous α-Syn into abnormal conformations by injection of α-Syn fibrils is critical for propagation of the α-Syn pathology. We have recently found that genetic deletion of A_2A_R reduced the α-Syn inclusions in both hippocampus and entorhinal cortex (Hu et al., 2016), suggesting that A_2A_R inactivation may abolish the seeding and possibly spreading effect of α-Syn fibrils. It would be important to explore whether caffeine might halt the seeding and possibly effect of α-Syn fibrils in future studies. Lastly, caffeine can also protect against β-amyloid-induced pathology and prion-induced pathology. Accordingly, chronic caffeine treatment reduces brain Aβ levels and protects against certain synaptic plasticity deficits (Alhaider et al., 2010; Costenla et al., 2010; Alzoubi et al., 2013) and cognitive deficits in aged AD transgenic (APPsw, Swedish mutation) mice (Arendash et al., 2006, 2009; Cao et al., 2009), and protects the neuronal cells against prion protein PrP (106-126)-induced cell death by inducing autophagy (Moon et al., 2014). Thus, caffeine is emerging as a potential novel strategy for preventing abnormal protein aggregation in neurodegenerative disorders.

### Caffeine selectively reverses α-Syn-induced defects in macroautophagy and CMA

Misfolded mutant A53T α-Syn fibrils are removed mainly via the autophagy-lysosomal pathway and ubiquitin proteasome system (UPS). As the UPS function is also closely associated with PD progress and A_2A_Rs can directly bind to and modulate UPS activity, caffeine's protection against α-synucleinopathy might be associated with modulation of UPS activity. However, A_2A_R activation has been shown to activate UPS activity (Chiang et al., 2009) and A_2A_R inactivation (by caffeine) likely impairs the removal of α-Syn, making the UPS less likely the target for caffeine-mediated neuroprotection. In agreement with this notion, we found no effect of caffeine on the UPS activity (as indicated by PSMC3 and proteasome 20S β6 protein levels) in the striatum. Consistently, soluble forms of excessive α-Syn has been shown to be efficiently degraded by the proteasome while aggregated α-Syn, as in the A53T α-Syn fibrils model of PD, is preferentially cleared by autophagy in cultured PC12 cells (Webb et al., 2003).

Autophagy is the cellular defense mechanism involving the lysosomal degradation of intracellular macromolecules or organelles in physiological conditions of differentiation, starvation and stress, such as the accumulation of abnormal protein aggregates (Menzies et al., 2017). Converging evidence from genetic, pathological and experimental studies suggests that in addition to the activation of autophagy of abnormal protein aggregates, these aggregates also impair autophagy pathway, contributing to PD pathogenesis. Among three dynamic pathways of autophagy, mutant α-Syn selectively impairs macroautophagy (an evolutionally conserved and constitutively active system that engulfs non-specifically cytosolic substrates after deformation of the lysosomal membrane). Thus, α-Syn aggregates impair overall macroautophagy by reducing autophagosome clearance (Tanik et al., 2013). Indeed, we detected marked defects in macroautophagy as evidenced by reduced LC3-II level and increased SQSTM1 level in α-Syn fibril-treated striatum, consistent with the previous studies using culture cells (Tanik et al., 2013). Importantly, we observed chronic caffeine treatment reverses these deficits by increasing level of LC3-II and decreasing level of SQSTM1 level, with no effect on beclin mRNA/ protein level. Together, these observations indicated that caffeine specifically modulates generation of autophagosomes and degradation of substrate in the whole autophagy processes, rather than influencing the initiation of macroautophagic flux.

Furthermore, chaperone-mediated autophagy (CMA) represents a highly specific process of autophagy that recognize a KFERQ-related targeting motif of soluble cytosolic proteins for degradation by a chaperone-complex involving the heat-shock cognate protein of 70 kDa (Hsc70). α-Syn is a substrate for the CMA, which recognizes a CMA-like recognition motif of α-Syn and translocates α-Syn to lysosomes for degradation. Specifically, phosphorylated α-Syn, A53T mutant (Cuervo et al., 2004), and dopamine-modified forms of the WT protein (Martinez-Vicente et al., 2008) lead to CMA dysfunction and may induce compensatory macroautophagy. The α-Syn-induced dysfunction of autophagy might be attributed to abnormally strong binding of α-Syn to the lysosomal receptor LAMP2A to prevent their own degradation. This led to defects in autophagosome axonal transport and consequently impaired lysosomal fusion and defect in lysosomal enzymatic activity. Notably, in contrast with the previous studies (Cuervo et al., 2004; Xilouri et al., 2009), we didn't observe any changes in Hsc70 in α-Syn-treated striatum, possibly due to the specific brain regions and temporal point we examined. The lack of Hsc70 changes in α-Syn-treated striatum may be due to the specific model of PD (intra-striatal injection of α-Syn fibrils) and limited temporal point examined (4 months after the treatment). However, we did observe the α-Syn-induced decreasing in LAMP2A and chronic caffeine treatment reversed this induction, indicating that caffeine may also modulate the CMA to influence α-Syn-induced pathological change. Further investigation into CMA in the various treatment time points and different PD models are required to clarify this issue.

Collectively, our finding demonstrate that chronic caffeine treatment selectively reverses the defects in macroautophagy and CMA induced by α-Syn by increasing LC3-II and LAMP2A and decreasing SQSTM1. To the best of our knowledge, the present study provides the first evidence that chronic caffeine treatment protects against mutant α-Syn-mediated neurotoxicity by re-establishing macroautophagy signals. This finding is in line with the view that caffeine induces a starvation response with induction of macroautophagy in yeasts (Winter et al., 2008; Saiki et al., 2011) and induce autophagy in various mammalian cell lines (Mathew et al., 2014; Hughes et al., 2017). In agreement with this view, caffeine reduces intra-hepatic lipid content and stimulates β-oxidation in hepatic cells with concomitant increase of autophagy and lipid uptake in lysosomes by down-regulation of mammalian target of rapamycin signaling (Sinha et al., 2014). Caffeine can also increase autophagy by the calcium-dependent activation of AMPK in skeletal muscle cells (Mathew et al., 2014) and confers protection against prion peptide-induced apoptosis in human SH-SY5Y cell line (Moon et al., 2014). The demonstration of caffeine modulation of autophagy in both PD models and liver diseases provide a common mechanism for epidemiological finding that human coffee consumption (and caffeine) is associated with reduced risk of developing PD and chronic liver disorders (Higdon and Frei, 2006).

Notably, chronic caffeine treatment in our study apparently did not affect autophagy in normal striatum, but selectively reversed the α-Syn-induced defect in macroautophagy and CMA. This suggests that chronic caffeine treatment may reestablish macroautophagy activity and broadly target a variety of protein aggregate with no-specific substrates in the brain. This selectivity of caffeine action is not clear and may be due to lack of specific effect of caffeine on hsc70. Furthermore, plasma concentrations of caffeine in our chronically caffeine-treated mice were at ≈2 μM range with preferential targeting at adenosine receptors. Whether caffeine's modulation of autophagy is mediated by blocking adenosine receptors need to be tested in future studies. However, caffeine can induce apoptosis through the enhancement of autophagy and the increased levels of LC3-II at concentrations >2.5 mM, a concentration likely acting at non-adenosine receptor targets such as phosphodiesterase inhibition and GABA receptor inhibition (Saiki et al., 2011). Thus, we speculate that caffeine modulation of autophagy is likely mediated by adenosine receptors as well as other pharmacological targets.

## Author contributions

J-FC, XR, WZ, YL: Conception and design; YL, ZZ, WZ, XR, ZH, XC, and FL: Experimental operations; YL, ZZ, WZ, and XR: Data collection; YL, XR, YG, J-FC: Analysis and interpretation; J-FC, YL, XR, WZ: Writing the article; J-FC, YL, XR, WG: Critical version of the article; J-FC: Overall responsibility.

### Conflict of interest statement

The authors declare that the research was conducted in the absence of any commercial or financial relationships that could be construed as a potential conflict of interest.

## Abbreviations

α-Syn: α-synuclein
A_2A_R: adenosine A_2A_receptor
PD: Parkinson's disease
pSyn: phosphorylated α-synuclein
AD: Alzheimer's disease
CMA: chaperone-mediated autophagy
LC3: microtubule-associated protein 1 light chain 3
LAMP2: lysosomal-associated membrane protein 2
SQSTM1/p62: sequestosome 1
ALP: autophagy-lysosomal pathway
GBA: glucosidase beta acid 1
GCase: glucocerebrosidase
IBA1: Ionized calcium binding adaptor molecule-1
TUNEL: TdT-mediated dUTP Nick-End Labeling
GFPA: glial fibrillary acidic protein.
