# Supplementary material for: Chronic Caffeine Treatment Protects Against α-Synucleinopathy by Reestablishing Autophagy Activity in the Mouse Striatum
Source: Front Neurosci. 2018 May 2;12:301. doi: 10.3389/fnins.2018.00301 (PMC5942142; doi:10.3389/fnins.2018.00301)
Supplement: Supplementary file 1 [file Image_1.PDF]

Supplement Figure for Luan et al. “Chronic caffeine treatment protects against α-synucleinopathy by reestablishing autophagy activity in the mouse striatum

Luan et al. Figure S1

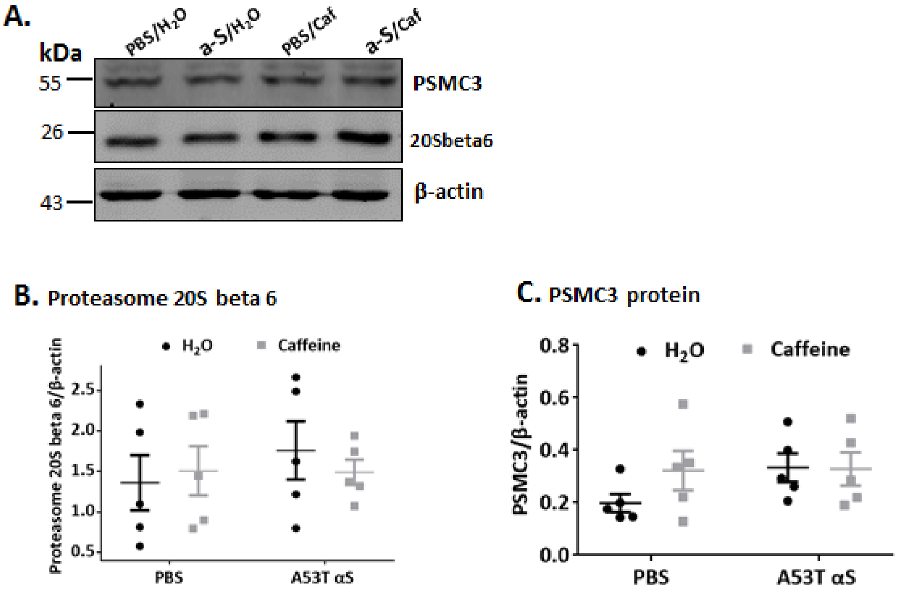

Supplementary Figure 1. Chronic caffeine treatment does not affect the UPS activity

C57B/L6 mice were bilaterally injected with PBS or A53T α-Syn fibrils into the striatum and treated with caffeine or water for four months. (A) Western Blot analysis of PSMC3 and Proteasome 20S β6. (B,C) Quantitative analyses of the protein levels of PSMC3 (B) and Proteasome 20S β6 (C). Chronic caffeine treatment did not alter the protein levels of PSMC3 and Proteasome 20S beta 6. n=5/group, $p>0.05$, two-way ANOVA.
