# Supplementary material for: Chronic Caffeine Treatment Protects Against α-Synucleinopathy by Reestablishing Autophagy Activity in the Mouse Striatum
Source: Front Neurosci. 2018 May 2;12:301. doi: 10.3389/fnins.2018.00301 (PMC5942142; doi:10.3389/fnins.2018.00301)
Supplement: Supplementary file 2 [file Image_2.pdf]

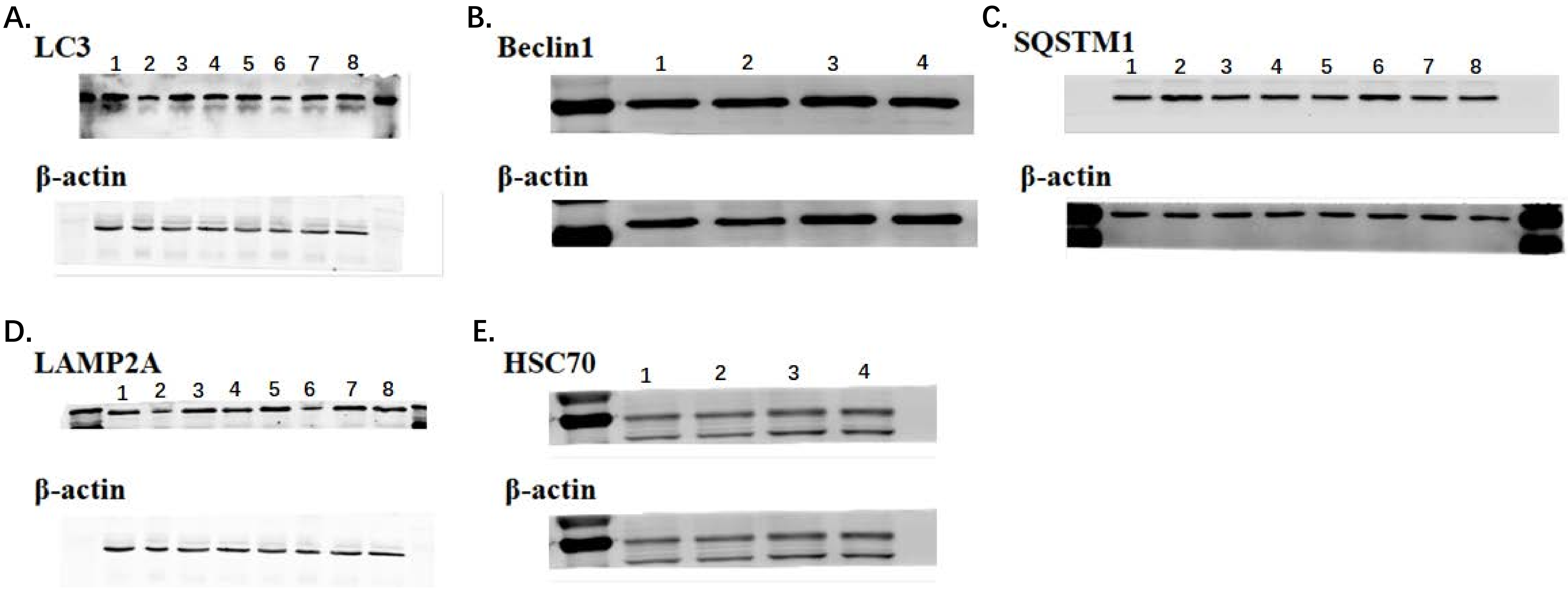

Figure S2: The original images of Western blot. For A, C and D: the lanes 1 and 5 correspond to the PBS/H_2O group, the lanes 2 and 6 correspond to the A53T/H_2O group, the lanes 3 and 7 correspond to the PBS/Caffeine group, the lanes 4 and 8 correspond to the A53T/Caffeine group. For B and E: the lane 1 is the PBS/H_2O group, the lane 2 is the A53T/H_2O group, the lane 3 is the PBS/Caffeine group, and the lane 4 is the A53T/Caffeine group.
